# Developing rejuvenation strategies and artificial flood mitigation plans for Indrani River: a case study

**DOI:** 10.1038/s41598-025-12439-z

**Published:** 2025-08-19

**Authors:** Shaz P. Muhammed, B. R. K. Holla, K. Balakrishna, Anish Kumar Warrier

**Affiliations:** 1 Department of Civil Engineering, Manipal Institute of Technology, Manipal Academy of Higher Education, Manipal, Karnataka 576104 India; 2 Centre for Smart Coastal Sustainability, Department of Civil Engineering, Manipal Institute of Technology, Manipal Academy of Higher Education, Manipal, Karnataka 576104 India; 3 Centre for Climate Studies, Department of Civil Engineering, Manipal Institute of Technology, Manipal Academy of Higher Education, Manipal, Karnataka 576104 India

**Keywords:** Artificial flooding, Rejuvenation strategies, Water quality assessment, Pollution, Waste management practices, Civil engineering, Environmental sciences, Environmental social sciences

## Abstract

Artificial flooding of rainwater is most common in urban areas due to various reasons, such as improper drainage systems, obstruction of natural drainage by building constructions, and encroachment of stormwater nallahs. Flash floods lead to significant losses, disrupt transportation, and cause inconvenience to the public. Udupi, characterized by its porous lateritic strata, undulating topography, and proximity to the sea, experiences artificial flooding during the peak monsoon season in its low-lying areas, primarily due to the overflow of the Indrani River, which is also a potential water resource for Udupi, Karnataka. Currently, the river faces significant challenges due to increasing anthropogenic activities. Revitalizing the Indrani River offers numerous benefits, including its potential use as a drinking water source during periods of water scarcity. This study aims to propose flood and stormwater management measures for the river catchment and to evaluate selected water quality parameters (pH, dissolved oxygen, and conductivity) at fifteen strategic locations along the river course. Higher conductivity observed at downstream stations is attributed to sewage discharge from urban settlements and a sewage treatment plant. The study suggests short-term measures such as targeted clean-up operations and stricter enforcement of pollution control regulations. Additionally, it recommends long-term strategies, including the development of a comprehensive river basin management plan, community engagement initiatives, and improvements to wastewater treatment infrastructure. To maintain the health of the Indrani River, this research emphasizes the importance of continuous monitoring and the implementation of integrated management practices.

## Introduction

Surface water resources and their quality are essential requirements for thriving biodiversity and sustainable development. Urban rivers are critical water resources for cities, providing water for drinking, irrigation, and other domestic uses. The quality of surface water bodies is closely linked to the ecological conditions of their watersheds and the pressures exerted by anthropogenic activities, which elevate pollutant concentrations. Industrial activities, rapid urbanization, and inadequate waste management practices have significantly deteriorated the quality and usability of water bodies, leading to severe pollution. Evaluating water quality is crucial for environmental management, particularly in river systems that are vital for drinking water supply, agriculture, and industry.

**Fig. 1:**
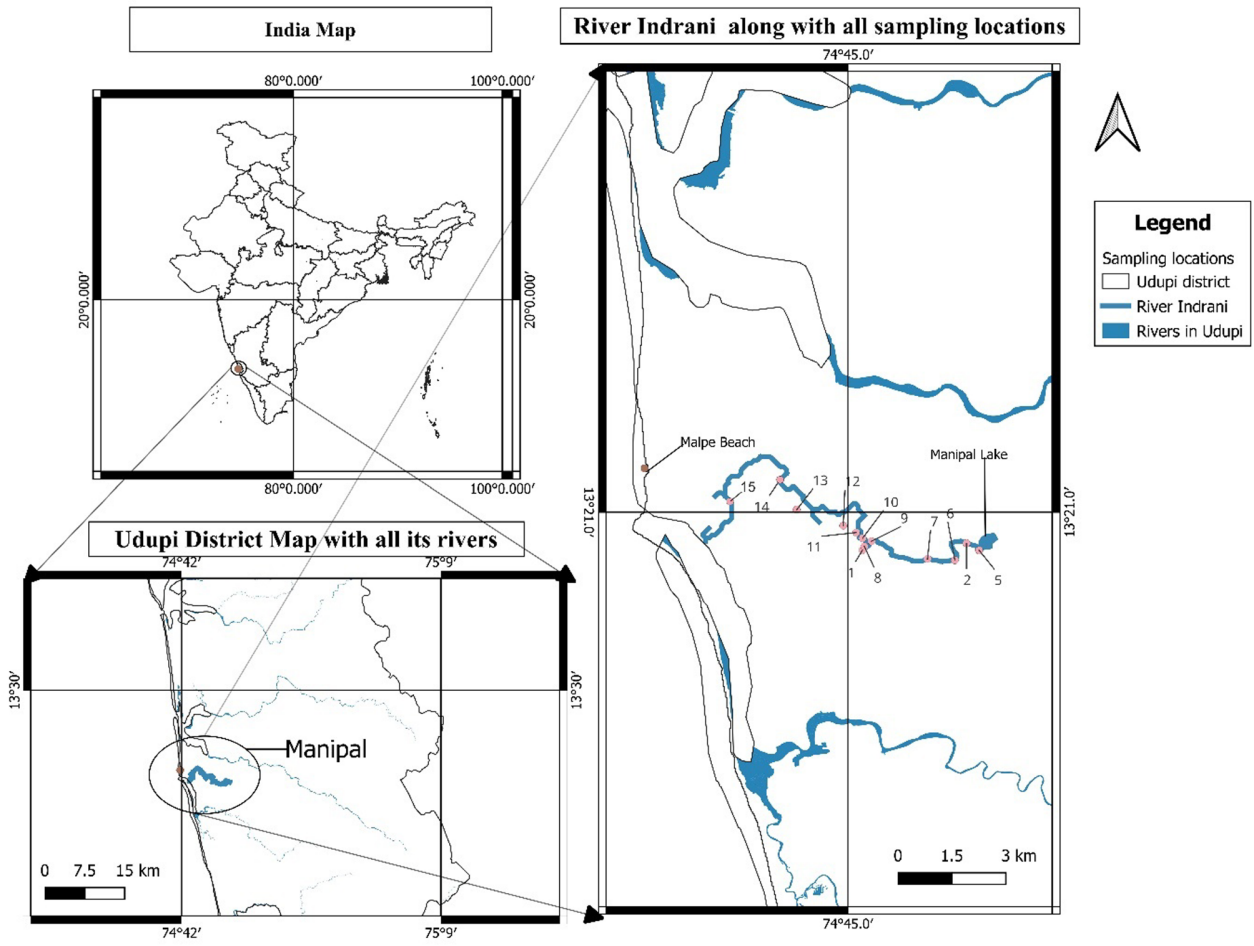
Study area map showing the Indrani River and the fifteen sampling locations along its course, including upstream, midstream, and downstream sites across regions with varying land use (urban, agricultural, and industrial zones). Map created using QGIS version 3.10.

In the Udupi district of Karnataka state, the Indrani River plays an important role in supporting ecosystems and local communities by aiding groundwater recharge and agricultue. The Indrani River, flowing through the center of Udupi city, originates from Manipal Lake, flows through Kalmady, and empties into the Arabian Sea at Malpe, Udupi, as shown in Fig. 1 (utilized QGIS (Quantum Geographic Information System) version 3.10, released in October 2019, for analysis. The software can be accessed at https://qgis.org). It spans a length of approximately 14 km long from its source to its mouth. The river serves as a potential freshwater source for drinking and agricultural purposes. In its upper reaches, the river is bordered by dense green cover, sustaining areca plantations and has a potential for attracting tourists to features such as waterfalls. Improper management has severely deteriorated water quality in the lower reaches, as evidenced by the findings of this study.

By examining physicochemical parameters at fifteen distinct points along the river channel, this study aims to comprehensively evaluate the water quality of Indrani River. The selected parameters include pH, dissolved oxygen (DO), dissolved oxygen saturation (DO%), electrical conductivity (EC), total dissolved solids (TDS), and salinity—all essential indicators of water quality. Water samples and site readings were collected from the strategically selected locations along the Indrani River, including upstream and downstream stretches, areas adjacent to agricultural and industrial activities, and regions with dense human settlements. The primary objectives of the analysis are to identify critical areas where water quality may be impaired and to assess spatial variations in water quality along the river course.

pH measures the acidity or alkalinity of water, which influences both the health of aquatic life and the solubility of nutrients and pollutants. Dissolved oxygen and the percentage saturation of dissolved oxygen (DO%) are critical for sustaining aerobic organisms; DO levels typically decrease with increasing water temperature and salinity and should ideally range between 6.5 and 8.5 mg/L in healthy freshwater ecosystems. Electrical conductivity (EC) measures the water’s ability to conduct an electric current and serves as a direct indicator of the concentration of dissolved salts and ions. Higher conductivity values generally reflect higher levels of dissolved substances. Total dissolved solids (TDS) represent the combined concentration of all organic and inorganic materials dissolved in the water, which can affect potability and overall water quality. Salinity impacts the osmoregulatory functions of aquatic organisms and can lead to poor health or mortality among native species, ultimately contributing to a decline in biodiversity^1^. By establishing a water quality profile for the Indrani River, this study aims to provide valuable insights into the current state of the river’s ecosystem, identify potential sources of pollution, and propose mitigation measures to maintain and improve water quality.

IS 10500 outlines a comprehensive methodology for managing and evaluating water quality, focusing on identifying potential risks and implementing appropriate mitigation measures. It emphasizes a systematic approach to water quality monitoring to safeguard public health and the environment. The standard specifies the recommended frequency, locations, and methods for water sampling to ensure data reliability and representativeness. It particularly recommends sampling at critical points, such as upstream and downstream of potential pollution sources, to effectively capture spatial variations in water quality.

IS 10500 includes a risk assessment framework comprising:

- Hazard Identification: Identifying potential pollution sources and hazardous substances.
- Risk Characterization: Assessing the likelihood and severity of adverse effects on human health and the environment.
- Risk Evaluation: Comparing measured water quality parameters against established standards and guidelines to evaluate the risk levels.

This data can aid environmentalists, policymakers, and water resources planners and managers in developing strategic plans to ensure the continued, uninterrupted flow of the Indrani River.

Water is fundamental to the survival of all species, including humans, and is a critical driver of global economic development. With a population exceeding 8.2 billion, the demand for water resources is rapidly increasing, and water scarcity has already become a reality in many regions worldwide. Pollution control and effective wastewater treatment are key components of water management strategies that help preserve the quality of sources supplying clean drinking water. Rapid urbanization, industrialization, and increasing population densities have introduced various forms of waste—including heavy metals, solid waste, plastic debris, and medical waste—into soils, sediments, and water bodies. Additionally, pollution arising from human, agricultural, and industrial activities, along with the accelerating impacts of global warming has contributed to the depletion of surface and groundwater resources^2^. These environmental changes pose severe threats to ecosystem functions and public health, demanding immediate attention and action. Human-induced heavy metal pollution enters the environment through multiple pathways, including urban runoff, livestock farming, mining activities, industrial operations, and the discharge of treated and untreated household and agricultural waste^3,4^.

Industries worldwide consume approximately 3,928 cubic kilometers of freshwater resources annually, according to the AQUASTAT database^5^. Industrial discharges have led to increased levels of organic and inorganic pollutants in rivers^4^. Heavy metals such as cadmium (Cd), nickel (Ni), iron (Fe), and arsenic (As), along with phenolic compounds, petroleum hydrocarbons, and sulfides, are difficult to completely remove from industrial effluents^6,7^. When these substances are released into the air, water, or soil, they disrupt biological systems, particularly affecting non-target organisms. Exposure to contaminated water supplies can result in serious health issues, including cyanosis, excessive sweating, reflex impairment, respiratory failure, cancer, reproductive disruption, and hypothermia.

The primary aim of urban drainage systems is to ensure the efficient discharge of runoffs into nearby water bodies. Reports suggest that over 90% of runoff flows directly into rivers in areas where both rainwater and sewage are diverted^8^. Centralized treatment methods often struggle to manage nonpoint source pollution because of its diverse nature. Such challenges are evident in major river systems; numerous investigations examining the physicochemical, biological, and toxicological characteristics of the Ganga River have demonstrated declining water quality and elevated levels of pollution^9^. Significant uncertainty remains regarding the physicochemical conditions of precipitation, runoff, and river systems, as well as the properties of particles deposited along riverbanks. Similarly, the probable fate of pollutants associated with particle-bound runoff after their release into waterways is poorly understood. Enhancing our understanding of these processes will enable more effective management and control of runoff pollution^8^.

The physicochemical characteristics of rivers, runoff, and rainwater vary significantly across the world. Studies have recorded pH values for rainwater, runoff, and rivers ranging from 3.77 to 7.12, 4.15 to 9.11, and 6.95 to 9.25, respectively; and electrical conductivity (EC) values ranging from 3.9 to 70.8 µS cm⁻¹ for rainwater, 24.1 to 2670 µS cm⁻¹ for runoff, and 153.4 to 1713 µS cm⁻¹ for rivers^8,10–17^. Variations in these parameters are often attributed to human activities such as the discharge of acid aerosols and wastewater from industrial and transportation processes^8,12^. During the eight-week nationwide lockdown implemented in response to the COVID-19 pandemic, the Ganga River showed evidence of rejuvenation and a considerable improvement in water quality. Reduced industrial effluent discharge, lower irrigation water extraction, and increased rainfall—which enhanced river flows and diluted existing pollutants were identified as key factors contributing to this improvement^18^. Given runoff water’s high pollutant concentrations, mobility, and bioavailability, it is crucial to focus on particles at the nano- and submicron scale. The study suggests that turbidity-to-total suspended solids (Tur/TSS) ratios can serve as useful indicators for evaluating the effectiveness of runoff pollution control strategies. These findings support the development of targeted measures to reduce runoff pollution and protect urban waterways^8^. Rainwater, characterized by its high oxidation-reduction potential (ORP) and low pH, readily leaches pollutants bound to particles. Once runoff is discharged into waterways, changing environmental conditions and complex physical, chemical, and biological processes within rivers may cause these pollutants to transition between particulate and dissolved phases^8^.

After consumption, 30–90% of a drug’s active ingredients are excreted through human urine. Despite wastewater treatment processes, approximately 90% of the active ingredients remain present in industrial effluents^19^. Pharmaceuticals such as antibiotics and painkillers can retain up to 90% of their activity even 28–40 years after manufacture^3,4,20^. Ternes (1998) emphasized that to restore river health, wastewater management issues must be addressed, adequate environmental flow releases must be maintained, and long-term, community-based approaches should be adopted to support pollution reduction programs^18^. In a related study, sampling points were selected away from wastewater effluent plumes, focusing on urban-industrial, rural-agricultural, estuarine, and mangrove areas along three rivers in southern India—the Kaveri, Thamiraparani, and Vellar rivers in Tamil Nadu. These rivers are significant sources of drinking water, and the detection of pollutants such as phthalate esters (HECs) raises concerns about potential human exposure through fish and water consumption. The investigation revealed the presence of phthalate esters in fish tissue, water, and sediment samples, indicating contamination of the aquatic environment^21^. Furthermore, the adoption of monitoring stations by the Central Pollution Control Board (CPCB) is expected to improve the implementation of river rehabilitation efforts. A study evaluating India’s National River Conservation Plan (NRCP) employed the Environmental Kuznets Curve model to analyze the relationship between income levels and pollution trends. The findings suggest that while the NRCP has significantly reduced chemical pollutants such as COD, it has had a negligible impact on untreated sewage and organic pollution, highlighting the need for improved pollution control strategies^22^.

To minimize phosphorus pollution, the Catchment Nutrient Balancing (CNB) method combines innovative solutions from both wastewater management and agriculture. This approach promotes better nutrient management practices on farms and incorporates technologies such as Polonite^®^ for extracting phosphorus from wastewater treatment facilities. The successful implementation of these strategies has been facilitated by effective collaboration among water companies, regulatory agencies, and community organizations. The CNB technique achieved a substantial yearly phosphorus load reduction of over 65% during a three-year pilot program in the Calthwaite Beck catchment in Cumbria, significantly surpassing the original target of 9%. Additionally, the river’s ecological classification improved from “poor” to “moderate“^23^.

The utilization of artificial intelligence (AI) and machine learning (ML) techniques for modeling water quality (WQ) has garnered increased attention from the scientific community in recent years. These methods have proven effective in WQ management, particularly by providing decision-relevant predictions to address challenges aligned with the United Nations Sustainable Development Goal for Clean Water and Sanitation (SDG 6)^24,25^. AI tools are commonly employed to improve cost-efficiency by estimating influent and effluent WQ parameters and optimizing wastewater treatment plant (WWTP) operations^25^.

Several hybridization strategies have been proposed to enhance the performance of deep learning (DL) models. These strategies include integrating multiple base models, as well as process-based, statistical-based, ML-based, attention-based, and transfer learning (TL) hybridization approaches. Research has shown that hybrid DL models outperform traditional DL models in terms of accuracy and fitting for WQ management. Integrating Internet of Things (IoT) systems, cloud computing, and DL models enables real-time monitoring, which is particularly useful for efficiently handling massive volumes of real-time data^25^. In one study, a hybrid machine learning approach was developed to predict the water quality of the Santiago River in Mexico using methods such as Support Vector Machine (SVM), Artificial Neural Network (ANN), and Adaptive Neuro-Fuzzy Inference System (ANFIS). Time series analysis and advanced clustering were employed to identify representative monitoring locations, eliminate redundant data, and enhance model performance. The ANFIS model demonstrated superior performance by achieving a root mean square error (RMSE) as low as 2.02 during training, outperforming conventional models trained without data selection^26^. Almost half of India’s population resides in the Ganga River Basin, which spans 11 states and is the primary focus of the Namami Gange Project. Similarly, China’s Yangtze River Protection Law addresses pollution control across the Yangtze River Basin, safeguarding a critical waterway that supports millions of people^27^. The release of pharmaceutical compounds into ecosystems over the recent decades without adequate treatment could have serious consequences for human health^28,29^. The review highlights that the world must develop targeted and hybrid solutions to eliminate these pharmaceutical residues. Pharmaceuticals found in wastewater can cause various detrimental effects on the environment and ecosystems. However, lack of suitable global data-gathering systems results in low-quality data and inadequate monitoring networks, posing a significant barrier to real-time WQ evaluation. Moreover, developing high-performance deep learning models for WQ prediction remains challenging due to the substantial computational resources required and the complexity of tuning numerous hyperparameters^25^.

## Research methodology

### General

The assessment of the Indrani River’s water quality was conducted using a structured research methodology comprising quantitative research methods, exploratory techniques, and a non-experimental design. This approach ensured a comprehensive and precise evaluation of water quality across various points along the river. The methodology involved systematically collecting samples and analyzing numerical data to quantify water quality parameters and identify patterns and relationships among sampling points across the upstream, midstream, and downstream sections.

As the study sought to thoroughly investigate the river’s water quality without predefined conclusions, it followed an exploratory research design. A non-experimental approach was chosen, eliminating the need for control groups or variable manipulation. Instead, the focus was placed on monitoring and evaluating the natural fluctuations in water quality parameters.

### Study area

The study area encompasses a 14-kilometer stretch of the Indrani River in Udupi district, Karnataka. It extends from its source at Manipal Lake (13.3525° N, 74.7881° E) to its mouth at the Arabian Sea near Malpe (13.3544° N, 74.7036° E) (Fig. 1). The low-lying areas along the river are prone to flash floods and waste accumulation. Pollution levels become acute beyond the 5-kilometer mark and reach severe levels near the river mouth.

### Sampling and analytical methods

This section describes the water sample collection procedures and analytical methods used in the study. Surface water samples were collected from the Manipal-Kalmadi River basin in February 2024. Sampling was conducted at 15 locations along the river, spanning from upstream to downstream areas. Sampling points were systematically selected at approximately 0.8 km intervals along the 14-kilometer river stretch, following the IS 3025 standards and CPCB (2017) guidelines for water quality monitoring. A combined approach involving systematic, stratified, and purposive sampling methods was employed. Systematic sampling involved collecting samples at regular intervals, primarily at each bridge along the river up to location 14. Sampling covered the entire river stretch—upstream, midstream, and downstream—based on geographic and environmental factors to ensure the representation of diverse riverine conditions.

Purposive sampling was employed to strategically select sampling locations on bridges, ensuring access to the middle of the river where water quality is more uniform and less influenced by localized factors such as stagnant water and surface runoff along the banks. Convenience sampling was used at location 15 due to inaccessibility between regions 14 and 15, as the river flows through dense forests and lacks nearby pathways. To address this, location 15 was selected at a bridge near the river mouth, capturing the diverse influences as the river connects to the Arabian Sea.

River water samples were collected using a polyethylene bucket secured with a nylon rope to prevent contamination from bridge materials. The water was transferred into pre-cleaned 1000 mL polypropylene (PP) bottles, which had been washed with ultrapure water and subsequently rinsed several times with river water prior to sampling to avoid contamination and ensure sample integrity. Water samples were collected from a depth of 1 to 3 m below the surface, considering river depth variations along the sampling path, which ranged between 2 and 5 m. This method minimized the influence of surface variability and ensured more representative sampling across different river sections.

Quality control measures were implemented throughout the study to assess contamination risks and ensure the reliability of the data analyzed. Water samples were collected and stored in high-grade polypropylene (PP) bottles to maintain sample integrity. Instruments were calibrated before analysis, and calibration curves were generated to ensure analytical accuracy. Three replicate analyses were performed for each sample. Ultrapure water (Milli-Q) and certified standards procured from Sigma-Aldrich were used throughout the experimental procedures. Reagent blanks were analyzed to verify the absence of contamination from reagents and consumables. Replicate analysis of samples and standards were conducted to evaluate precision, while known concentrations of standards were used to assess accuracy.

The Bureau of Indian Standards (BIS) establishes acceptable and permissible limits for several key water quality parameters, including: pH (6.5–8.5), turbidity (≤ 1 NTU), total dissolved solids (TDS ≤ 500 mg/L), and hardness (≤ 200 mg/L).  These limits were used as the criteria in interpreting the obtained data^30^.

During seasonal sampling, physicochemical parameters of water samples—including temperature (T; °C), pH, electrical conductivity (EC; µS cm⁻¹), dissolved oxygen (DO; mg L⁻¹), total dissolved solids (TDS; mg L⁻¹), and salinity—were measured on-site using HACH multi-parameter instruments available at the Environmental Research Laboratory (ERL), Department of Civil Engineering, Manipal Institute of Technology (MIT), Manipal. The instrument consists of three probes: a pH probe, a conductivity probe, and a DO probe. The conductivity probe measures the electrical conductivity of the samples and provides readings for TDS and salinity. Before measurements, the pH and conductivity probes were calibrated using HACH-supplied buffer and standard solutions. The pH probe was calibrated with standard buffer solutions at pH 4.01, 7.01, and 10.02, while the conductivity probe was calibrated with a NaCl solution of 1000 µS cm⁻¹. The measurement precision of the instrument was ± 0.3 °C for temperature, ± 0.01% for pH, ± 0.1 mg L⁻¹ for DO, ± 0.5% for EC, ± 0.4 mg L⁻¹ for TDS, and ± 0.1% for salinity. For each parameter, readings were recorded only after the instrument achieved stabilization.

**Fig. 2:**
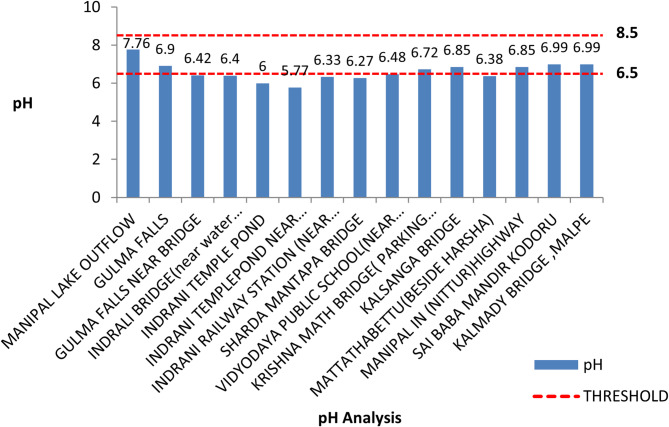
pH values recorded at each sampling location along the Indrani River, highlighting spatial variations relative to the acceptable pH threshold range (6.5–8.5).

**Fig. 3:**
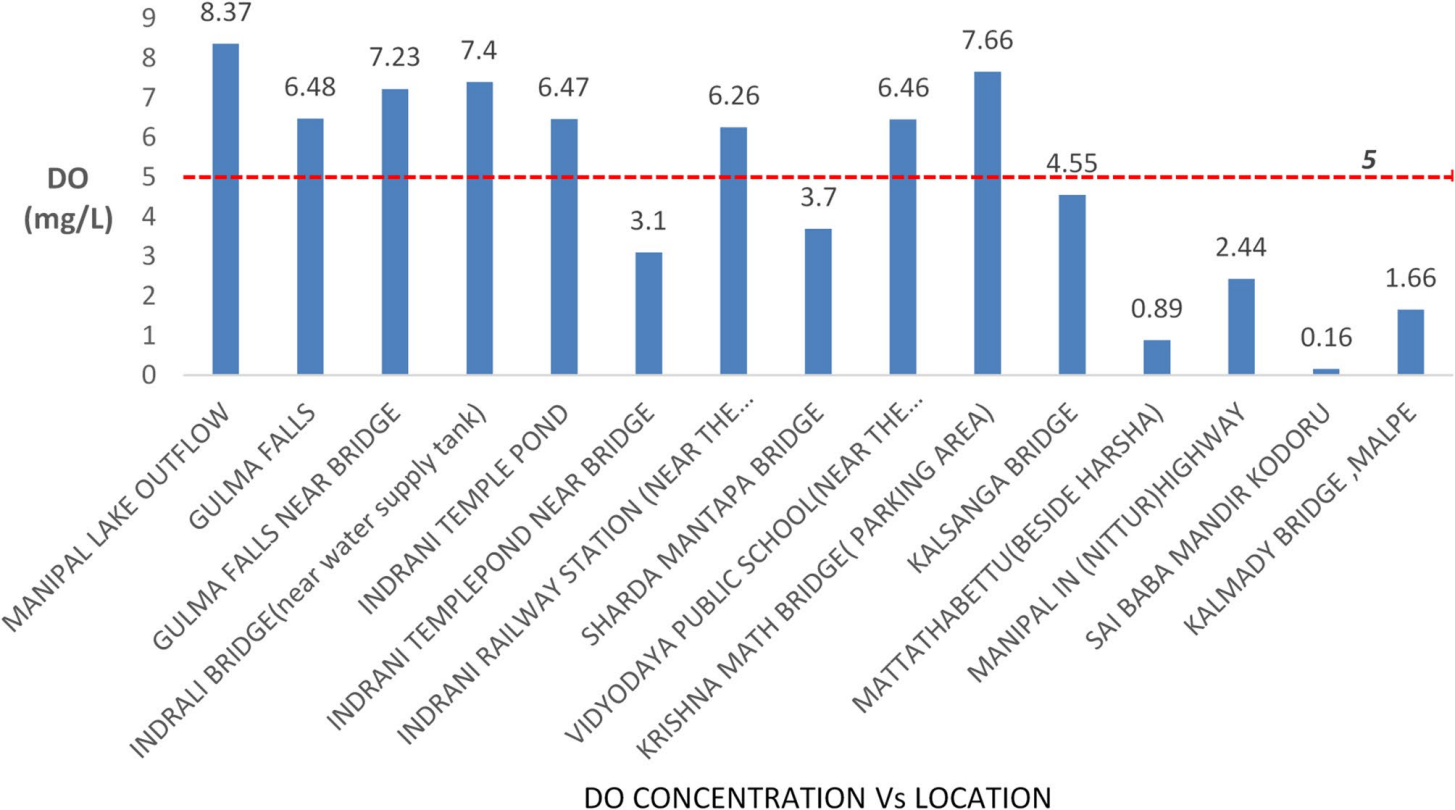
Dissolved oxygen (DO) values recorded at each sampling location along the Indrani River, with reference to the threshold value of 4 mg/L for maintaining healthy aquatic conditions.

**Fig. 4:**
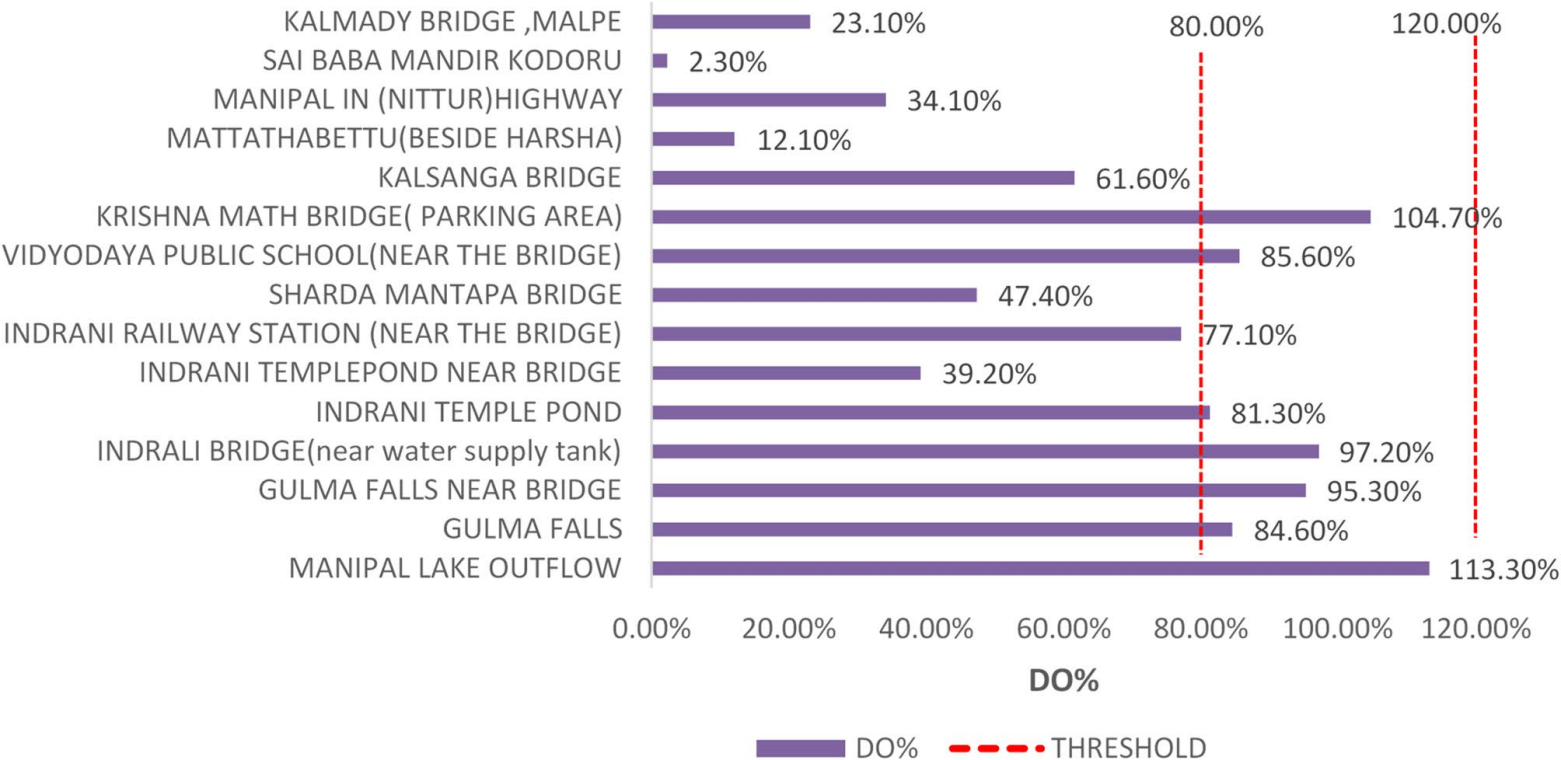
Dissolved oxygen percentage (DO%) values recorded at each sampling location along the Indrani River, highlighting variations relative to the acceptable threshold of 80% saturation for healthy aquatic ecosystems.

**Fig. 5:**
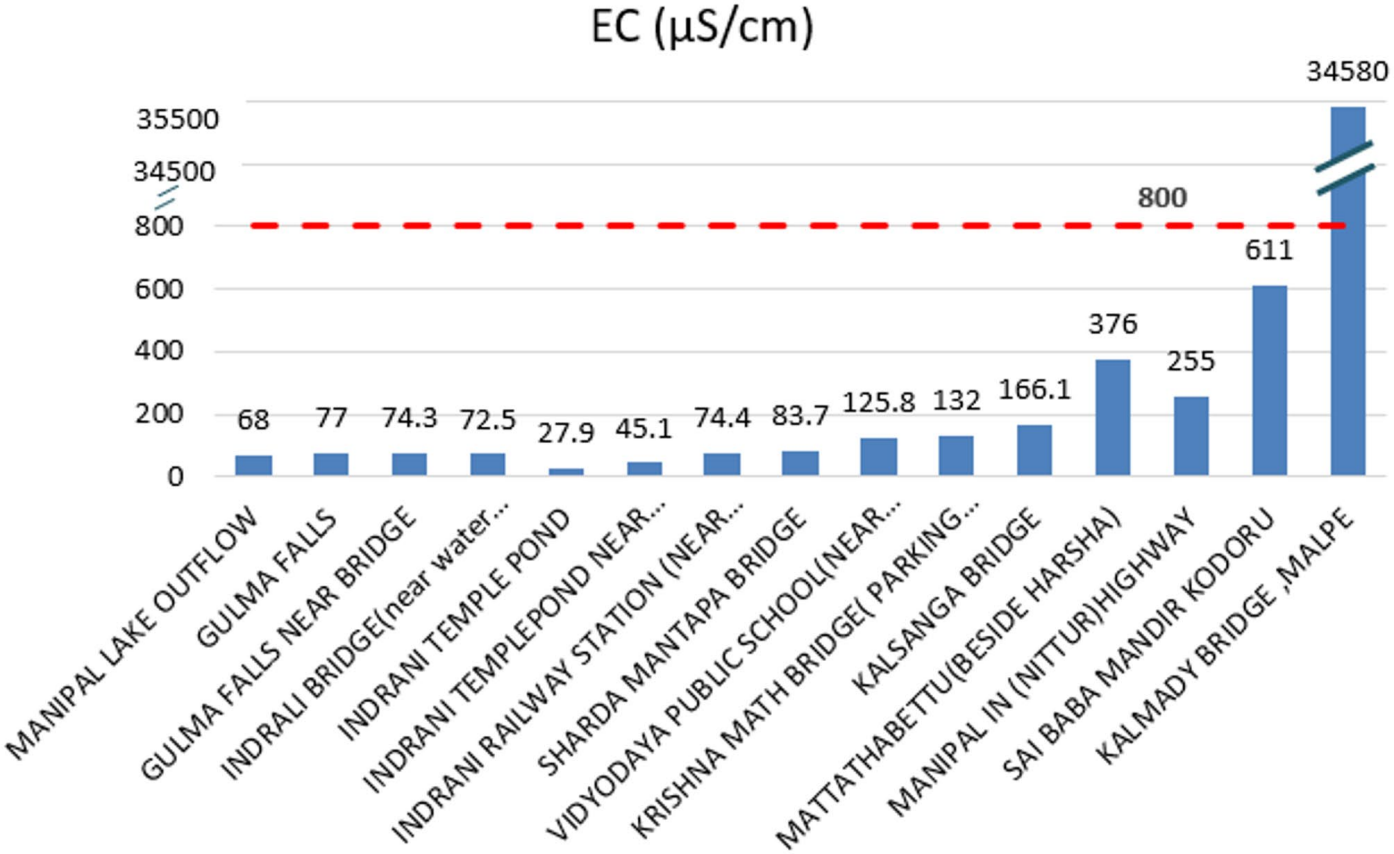
Horizontal bar chart showing electrical conductivity (EC) values recorded at each sampling location along the Indrani River, highlighting spatial variations and identifying locations exceeding the acceptable threshold of 800 µS/cm.

**Fig. 6:**
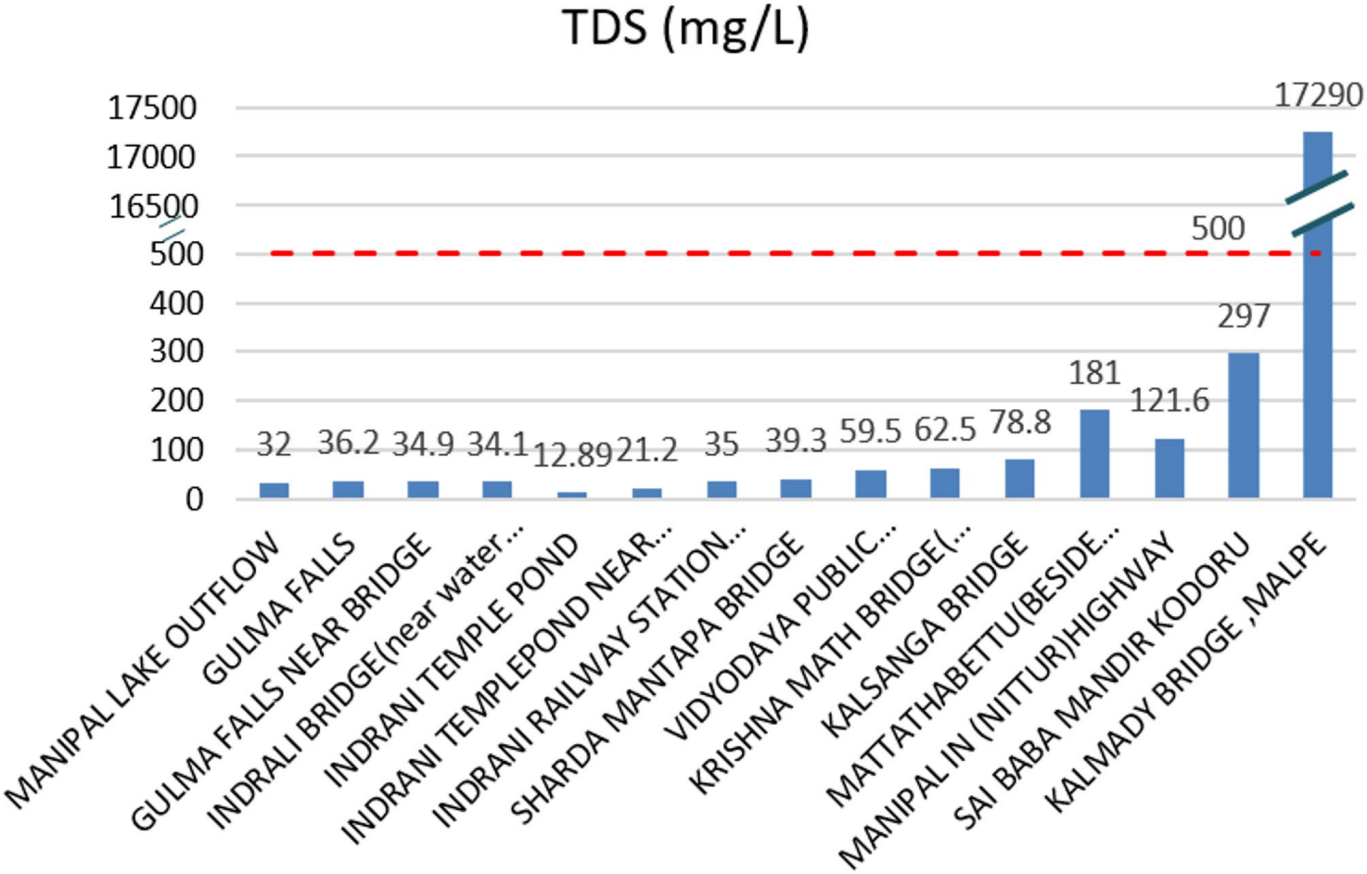
Total dissolved solids (TDS) values recorded at each sampling location along the Indrani River, highlighting spatial variations and identifying critical increases near the river mouth relative to the acceptable threshold of 500 mg/L.

**Fig. 7:**
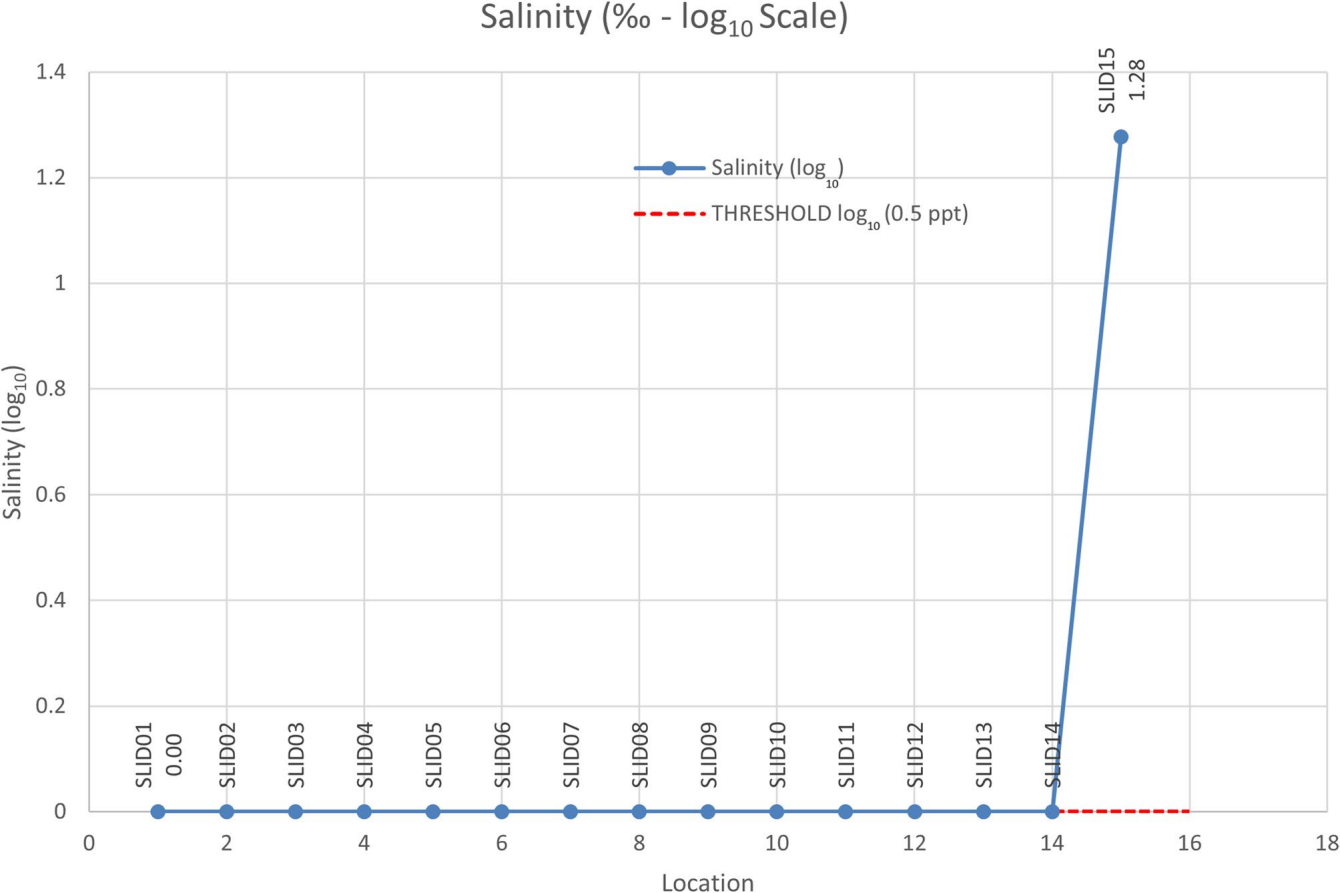
Salinity values (log₁₀ scale) recorded at each sampling location along the Indrani River, highlighting significant deviation at the river mouth due to seawater intrusion relative to the acceptable salinity threshold of 0.5 ppt.

Statistical analysis was conducted using Microsoft Excel 2019 (Version 16.0.10417.20030)) to calculate the maximum and minimum values and to prepare charts for all physicochemical parameters of the Indrani River water samples. The results were then color-coded based on threshold values: values within permissible limits were marked in green, while deviations were highlighted in yellow (moderate risk) and orange (high risk). Color categorization was performed using Excel’s conditional formatting algorithms. Correlation analysis was also carried out to examine the relationships between variables, aiding in the identification of potential influencing factors. All graphical representations, from Figs. 2, 3, 4, 5, 6 and 7, were generated using Microsoft Excel software. Additionally, a 3D model of the proposed action plan was developed using SKETCHUP PRO 2021 software, designed with appropriate dimensions to support visualization of the interventions.

## Results and Discussion

The results and discussions based on the analyzed data are presented below, as summarized in Table 1.

**Table 1:**
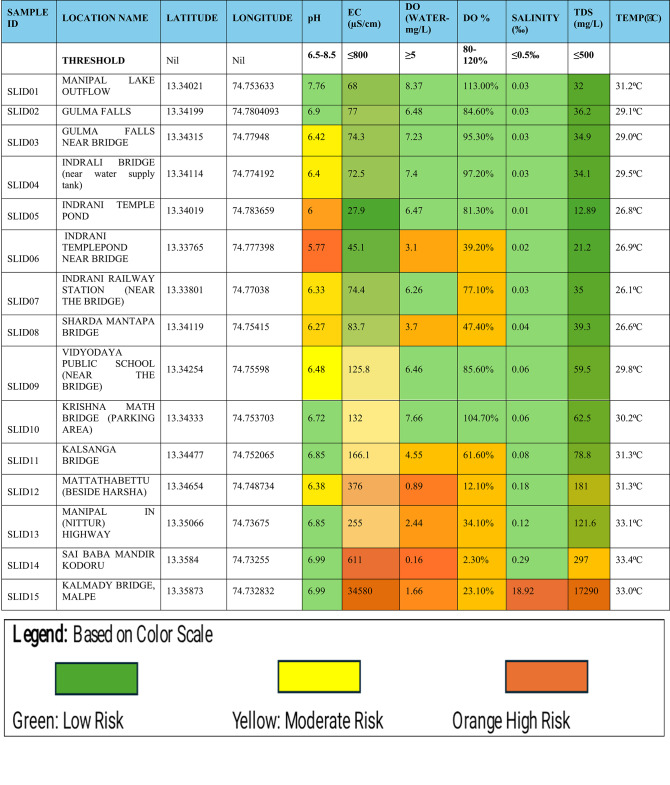
Risk classification of water quality parameters for the Indrani river based on color-coded threshold compliance, indicating low-, moderate-, and high-risk locations.

### Data analysis and results

#### pH analysis

The pH analysis along the Indrani River showed that pH levels fluctuated around a median value of 6.19. As illustrated in Fig. 2, a dip in pH levels was observed at the first six locations. The acceptable pH range for natural waters is typically from 6.5 to 8.5, and it was noted that 8 out of 15 locations (~ 55%) recorded pH values outside this threshold. An increase in pH values was observed from location 05 onwards, likely due to the confluence of multiple streams and a rise in salinity as the river approached the ocean. The lowest recorded pH value was 5.77 at location 05, indicating acidic conditions likely resulting from rainfall-derived acidity originating from springs at the river’s source.

At the starting point, Manipal Lake (the source of the Indrani River and the highest elevation among all sampling points), a pH of 7.76 was recorded. The observed alkalinity at this location may be attributed to the consumption of carbon dioxide by algal photosynthesis, which is supported by the higher dissolved oxygen levels measured concurrently. Locations 01 and 02, with pH values of 6.9 and 6.42 respectively, are likely influenced primarily by groundwater contributions. As the river widens and progresses toward the sea, the pH values tend to normalize. Lower pH values at locations 08 and 09 may be attributed to high levels of waste disposal from surrounding residential areas and reduced water flow, possibly due to the construction of a check dam. It is recommended that seasonal variations in pH and other water quality parameters be monitored throughout the year to better understand the temporal dynamics of water quality and to assess potential risks to river health.

#### Dissolved oxygen analysis

Dissolved oxygen (DO) levels varied significantly across different locations along the Indrani River. The highest DO value (8.37 mg/L) was recorded at location 01, which exhibited relatively clean conditions and supported some biodiversity. According to WQ standards, the permissible threshold for DO is 5 mg/L or higher. As shown in Figs. 3, 4 and 6 out of 15 locations (40%) recorded DO levels below the permissible limit, indicating potentially serious environmental degradation if remedial actions are not taken. A general decline in river ecosystem health was reflected in the observed decrease in DO values across several locations. The lowest DO value (0.16 mg/L) was observed at location 14, characterized by stagnant water conditions primarily due to the discharge of untreated municipal sewage. Black patches indicating waste accumulation were evident at the site during sample collection. Similarly, location 12 exhibited the second-lowest DO value (0.89 mg/L), attributed to the discharge of inadequately treated effluent from a nearby sewage treatment plant.

Acceptable DO levels (greater than 6 mg/L) were observed at the first five locations, indicating relatively unpolluted upstream conditions. However, locations 05, 06, and 08 exhibited DO levels lower than the threshold, likely due to reduced flow rates and the accumulation of solid waste and plastics. At location 11, DO values were close to the threshold, influenced by high pollution levels within the urban center, making the river more vulnerable to waste disposal. At the Nittur highway crossing, a DO value of 2.44 mg/L was recorded, likely due to reduced flow and wastewater accumulation; structural damage to aqueducts was also noted, impeding water movement. A low DO value of 1.66 mg/L at the river mouth (location 15) can be attributed to the influx of saline seawater, which typically holds less oxygen than freshwater. Dissolved oxygen percentage (DO%) represents the saturation level of oxygen in water relative to its maximum holding capacity, with acceptable DO% values typically exceeding 80%. The observed patterns in DO and DO% underline the significant anthropogenic pressures on the river’s ecological health.

#### Electrical conductivity analysis

The electrical conductivity (EC) values measured along the Indrani River were generally found to be within the acceptable threshold of less than 800 µS/cm, except at location 15, where an exceptionally high value of 34,580 µS/cm was recorded. This sharp increase is likely due to the mixing of river water with saline seawater at the river mouth. A reading of 611 µS/cm was detected at location 14, suggesting the accumulation of inorganic salts and chemicals in the water. After location 8, a progressive increase in EC values was observed at each subsequent site, indicating worsening WQ likely associated with rising population and urban pollution, as illustrated in Fig. 5. Location 12 exhibited a very foul odour, and improper treatment of sewage water may have contributed to the elevated EC readings. The lowest EC value of 27.9 µS/cm was recorded at location 5, reflecting relatively pristine conditions. Elevated EC values were also observed at three additional locations beyond location 11, primarily due to deteriorating water conditions caused by pollution inputs. These trends highlight a significant risk of declining water quality and emphasize the urgent need for targeted management and pollution control measures.

#### Total dissolved solids (TDS) analysis

The threshold value for total dissolved solids (TDS) in river water should not exceed 500 mg/L to maintain optimal river health. At most locations along the Indrani River, TDS levels remained below this threshold, with the notable exception of location 15, where a very high TDS value of 17,290 mg/L was recorded, as shown in Fig. 6. Although TDS levels at intermediate locations remained within acceptable limits, some readings approached the threshold, indicating potential future risks if pollution inputs are not controlled. TDS values progressively increased downstream, reaching a peak at the river mouth, likely due to the mixing of saline seawater with river water. At location 14, dark circular patches, stagnant water, and the accumulation of plastic waste, including numerous discarded water bottles, were observed. Although the TDS value at location 14 remained within permissible limits, the physical condition of the site suggests increasing pollution pressure. The lowest TDS value (12.89 mg/L) was recorded at location 05, likely due to the influence of spring water inflows at that site. A sharp increase in TDS values was observed from location 12 onward, corresponding with deteriorating water quality and visible signs of pollution. These results highlight the emergence of pollution hotspots along the river channel, underlining the urgent need for targeted interventions to maintain the ecological health of the Indrani River.

#### Salinity analysis

Salinity values were plotted on a log10 scale (Fig. 7) to better visualize significant deviations among sampling locations. The acceptable threshold for salinity in river water is less than or equal to 0.5 parts per thousand (ppt). All locations recorded salinity values within the acceptable range except for location 15, where a substantial increase was observed due to seawater intrusion. The highest salinity value (18.92 ppt; log10 value: 1.27) was recorded at location 15. At location 14, a salinity value of 0.29 ppt (log10 value: − 0.537) was measured, which, although still within permissible limits, was approaching the threshold value of 0.5 ppt (log10 value: − 0.30). This rise in salinity at location 14 may reflect increasing pollution pressure and the influence of organic and inorganic contamination near the downstream reaches of the river.

### Discussion

Descriptive statistics for parameters such as pH, DO, EC, TDS, salinity, and temperature were obtained from water samples analyses and represented through individual charts, as discussed above (Figs. 2, 3, 4, 5, 6 and 7). Threshold values for each parameter were indicated within the charts to facilitate direct interpretation and assessment. The charts were analyzed to identify spatial trends and to evaluate differences in water quality parameters between sampling locations using descriptive statistical methods. The results were subsequently compared to established water quality standards to assess risk levels and to identify critical areas of concern along the river. 

Several challenges were encountered during data collection and analysis. Data variability was notable, with marked changes observed after location 8 and spatial variations from upstream to downstream sections. Navigation through dense forests and uneven terrain also posed difficulties at various points along the river, as there were no proper banks or stable paths to access sampling sites. Seasonal variations were not captured in this study, limiting the assessment of temporal risk fluctuations. Pollution-related parameters generally exhibited higher values in urban stretches. As most sampling activities were conducted between 7:00 am and 3:00 pm during the daytime, the influence of diurnal temporal variation was minimized. Natural factors such as sediment transport, erosion, and biological activity were evident in the river’s first and second stretches, resulting in observable changes in water quality. In contrast, anthropogenic factors predominated in the third and fourth stretches, where the river flows through urbanized areas. 

Onsite pH measurements across the 15 sampling locations in February 2024 ranged from 5.77 to 7.76, with an overall average value of 6.60. The highest pH value (7.76) was recorded at location 01, while the lowest value (5.77) was observed at location 06, indicating slightly acidic conditions outside the acceptable threshold range (6.5–8.5). Only 7 out of 15 locations complied with the threshold range for pH. Water temperature across locations ranged from 26.1 °C to 33.4 °C. Electrical conductivity (EC) values ranged from 26.9 to 611 µS/cm, remaining below the BIS-prescribed threshold of 800 µS/cm. However, values at locations 12, 13, and 14 approached the threshold, signaling rising pollution levels and the need for prioritized mitigation efforts. Dissolved oxygen (DO) concentrations ranged from a minimum of 0.16 mg/L at location 14 to a maximum of 8.37 mg/L at location 01. Six out of fifteen locations recorded DO levels below the permissible threshold of 4 mg/L. A consistent decline in DO values was noted downstream from location 11 onwards, reflecting deteriorating water quality and increasing pollution.

Total dissolved solids (TDS) concentrations ranged from 12.89 mg/L at location 05 to 297 mg/L at location 14, remaining within the 500 mg/L threshold. A gradual rise in TDS values was observed starting from location 09, indicating progressive pollution accumulation. Salinity values remained below the 0.5‰ threshold at all locations except location 15, where a value of 18.92 ppt was recorded, attributed to seawater intrusion. Salinity values also showed an increasing trend beginning at location 11. Locations 12, 13, 14, and 15 showed the most pronounced deviations from threshold standards across multiple parameters, identifying them as critical pollution hotspots. Among these, location 14 was categorized as a high-risk site, while locations 11, 12, and 13 were classified under moderate-risk zones, as summarized in Table 2. Immediate interventions, such as pollution source control and habitat restoration, are recommended for high-risk zones identified along the lower stretches. From locations 03 to 09, a relative dip in water quality was observed, possibly linked to agricultural runoff and residential wastewater discharge. The observed increase in EC and TDS values from location 11 onward reflects the cumulative impact of urban pollution and highlights the urgent need for river rejuvenation efforts in the lower stretches of the Indrani River.

**Table 2:** Water quality parameters of the Tungabhadra River^31^.

	Temperature (°C)	Total hardness (mg/L)	TDS (mg/L)	DO	pH
Values	28.15–31.65	109–160	176.75–271	5.9–8.1	7.24–8.2
Comments	Fair	Acceptable	Below acceptable limit	Suitable for drinking	Acceptable

The color-coded risk assessment for each water quality parameter is presented in Table 1. Green cells indicate compliance with the acceptable limits prescribed by the Bureau of Indian Standards (BIS) for river water quality. Color scaling toward orange signifies an increasing risk of pollution, with dark orange representing high-risk conditions and lighter shades indicating moderate-risk zones. The risk chart enables the identification of low-, moderate-, and high-risk locations across the river channel. Among the five parameters analyzed, dissolved oxygen (DO)—a critical indicator for the health of aquatic organisms and suitability for human use—exhibited high-risk levels at 7 out of 15 locations, representing approximately 40% of the river stretch. These findings highlight the need for urgent attention and targeted mitigation measures to restore river health in the most affected zones.

A comparison was made between the Indrani River and the Tungabhadra River (Table 2), which flows through the states of Karnataka and Andhra Pradesh. The basic water quality parameters of the Tungabhadra River were found to be within the acceptable range^31^. Indian history and culture are intricately linked to the Tungabhadra River, particularly through its association with Hampi—a UNESCO World Heritage Site and a key centre of the Vijayanagara Empire. In contrast, the Indrani River exhibited greater variation in pH levels and more pronounced depletion of dissolved oxygen concentrations. TDS values also demonstrated wider variability in the Indrani River compared to the relatively stable levels observed in the Tungabhadra River. Both rivers, however, exhibited signs of anthropogenic influence, including the discharge of untreated domestic sewage and industrial effluents, underscoring the widespread impact of human activities on river water quality.

**Table 3:** Ranking of proposed action plans based on their calculated cost-benefit ratio (CBR) values for prioritizing interventions along the Indrani River.

CBR ratio			
Sl no	Long term plans	CBR	Priority by CBR
1	Developing STP infrastructure (Nanomembrane Technology)	0.25	4
2	Monitor water quality periodically	3.5	1
3	Widen and maintain river channels	0.44	2
4	Create coastal reservoirs	0.39	3

**Fig. 8:**
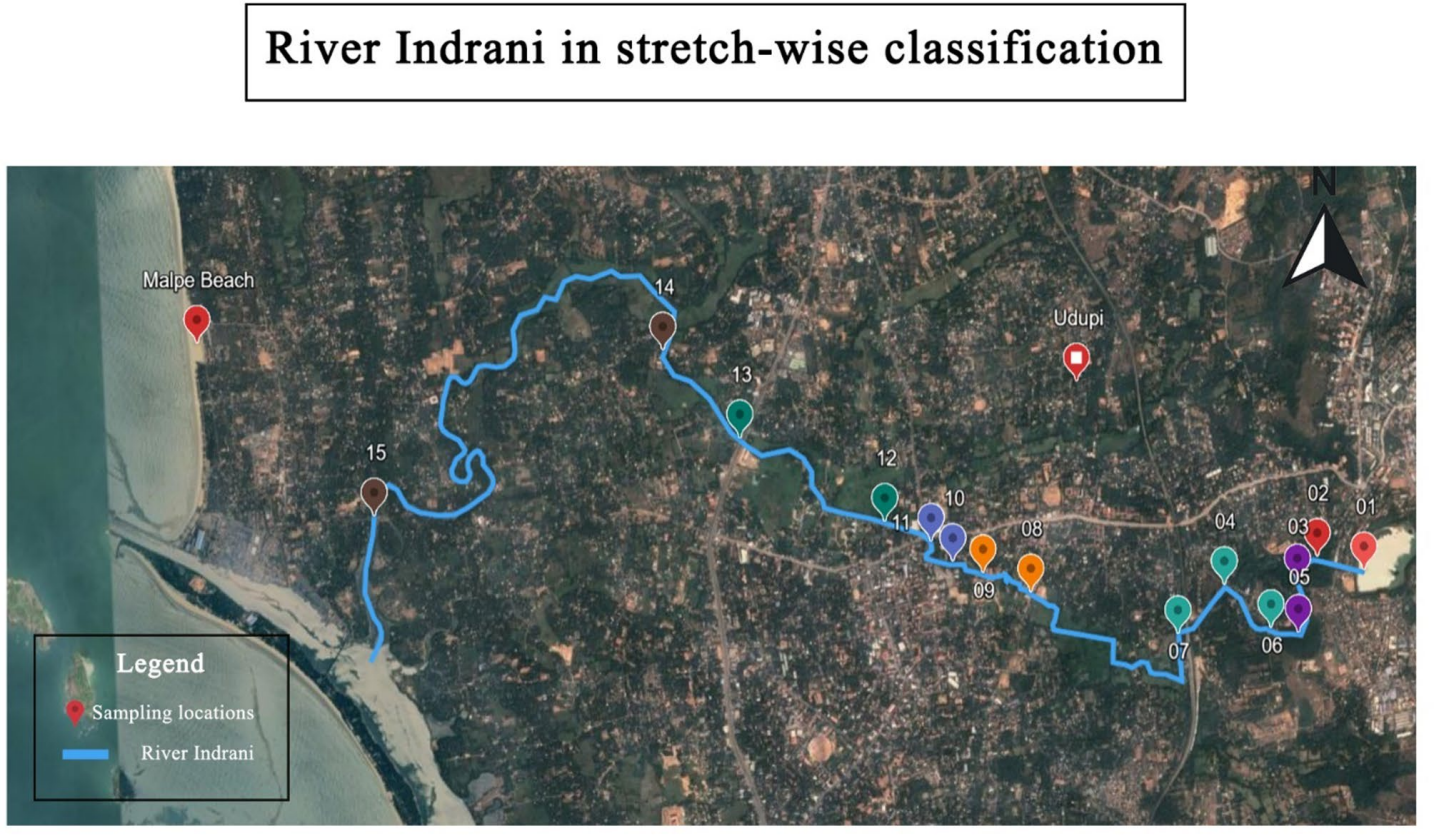
Study area map with stretch-wise classification (Software used: Google Earth https://earth.google.com/web/search/udupi/@13.3318086,74.74700955,47.98053677a,18096.42392957d,35y,0 h,0t,0r/data=CiwiJgokCd3esl3ofTRAEZQBoSBT0DLAGQ2rpCnIf0pAISpcPwFvTEjAQgIIAUICCABKDQj___________8BEAA).

### Action plans for rejuvenating the Indrani River

The present condition of the Indrani River indicates moderate contamination, particularly across downstream segments. To facilitate a target response, the entire river stretch was classified into seven segments based on distance to assess pollution status, as shown in Table 4 and visually represented in Fig. 8. Figure 8 offers a detailed visual representation created by the authors using Google Earth software and is accessible via the following URL: https://earth.google.com/web.

**Table 4:** Grouping of sampling locations along the Indrani River into distinct stretches based on geographic, environmental, and land-use characteristics.

Stretch #	Locations
Stretch 1	01, 02
Stretch 2	03, 04
Stretch 3	05, 06, 07
Stretch 4	08, 09
Stretch 5	10, 11
Stretch 6	12, 13
Stretch 7	14, 15

At each sampling location, two water samples were collected for laboratory analysis, while onsite measurements were conducted using a HACH multiparameter probe. Out of the seven classified stretches, four were found to be significantly impacted by pollution. Several residential areas were observed discharging untreated sewage directly into the river, contributing to ongoing water quality degradation. The study aimed to identify pollution hotspots, defined as locations where water quality parameters exceeded permissible limits. Among the 15 locations surveyed, 7 were identified as hotspots based on dissolved oxygen (DO) levels falling below the acceptable threshold of 5 mg/L. Furthermore, the practice of discharging agricultural runoff directly into the river has further exacerbated pollution levels, particularly in downstream segments.

Additional environmental stressors were observed, including land encroachments along the riverbanks, which were documented at various locations, including 07–11 and 14, plastic waste accumulation, particularly at locations 11, 13, and 14, threatening aquatic life and degrading overall river health. In addition, the collapse of certain infrastructure at locations such as 13 has hindered natural water flow, leading to waste accumulation and resulting in foul odours in the surrounding areas. Moreover, issues with the functionality of sewage treatment facilities were noted, with significant problems observed at location 12, resulting in strong and unpleasant odors in the surrounding area. These combined stressors further exacerbate the degradation of the Indrani River’s ecological condition and highlight the urgent need for coordinated restoration efforts.

These observations underscore the urgent need for comprehensive measures to address the pollution and environmental degradation affecting the Indrani River, highlighting the need for sustainable management practices and active community engagement to preserve this vital natural resource. To support decision-making and real-time monitoring, a consolidated platform for up-to-date information on water quality, quantity, and ecosystem conditions is provided by the Karnataka Water Resources Information System (KWRIS). This system offers the potential for effective monitoring of the Indrani River’s salinity, pH, and dissolved oxygen levels, thereby supporting timely interventions to address challenges such as stagnant water conditions and rising pollution levels.

### Short-term plans for Indrani river restoration

#### Immediate pollution control

- *Identification of Hotspots*: Conduct detailed surveys to locate both point and non-point sources of pollution, including sewage disposal points, agricultural runoff zones, and industrial discharges, particularly from fish processing units.
- *Effluent Treatment Plant (ETP) and Sewage Treatment Plant (STP) Inspections*: Ensure that all STPs in the river basin are fully functional, equipped with operational effluent treatment systems, and compliant with environmental standards.
- *Strengthening Enforcement*: Intensify the enforcement of environmental regulations to prevent illegal dumping and untreated discharges into the river.

#### Water quality monitoring

- *Establishment of Monitoring Stations*: Set up permanent water quality monitoring stations at identified pollution hotspots along the river.
- *Reliable Data Collection Systems*: Implement a robust and continuous data collection system to detect trends, assess pollution levels, and prioritize areas needing immediate intervention.

#### Community engagement

- *Awareness Campaigns*: Launch public education campaigns through skits, murals, posters, social media outreach, and seminars to highlight the importance of river conservation and the impact of pollution.
- *Community Involvement*: Engage schools, local communities, and NGOs in regular river clean-up activities and conservation workshops.
- *Incentivized Participation*: Utilize CSR (Corporate Social Responsibility) funds collected from regional industries to incentivize public participation in conservation initiatives.
- *Creation of a Supervised Community Group*: Establish a government-supervised community group or club responsible for addressing new challenges, securing funding, and receiving guidance from advisory boards comprising experts from the government and private sectors.
- *Policy Proposals*: Advocate for stricter enforcement of pollution control regulations, promotion of sustainable industrial practices, and dedicated funding allocations for river restoration efforts.

#### Waste management improvements

- *Enhancement of Waste Collection Systems*: Upgrade and streamline waste management infrastructure in surrounding areas to prevent waste entry into the river.
- *Promotion of Composting*: Encourage the composting of biodegradable waste at the community level through awareness programs and incentives, reducing the organic load entering the river.

### Long-term plans for Indrani river restoration

#### Infrastructure development

- *Upgrading Sewage Treatment Plants (STPs)*: Collaborate with government rejuvenation programs to develop and modernize STPs to handle increased wastewater capacity and improve operational efficiency for future demands. For instance, under the Namami Gange initiative, Varanasi’s sewage treatment capacity increased from 100 MLD in 2014 to 420 MLD by 2024^27^.
- *Rainwater Harvesting Initiatives*: Implement widespread rainwater harvesting systems to reduce surface runoff, recharge groundwater, and alleviate pressure on river systems.

#### Urban planning and management

- *Zoning Regulations*: Introduce and enforce zoning policies to prevent unregulated development near riverbanks.
- *Floodplain Restoration and Retention Zones*: Rehabilitate natural floodplains and establish water retention zones, similar to successful interventions along the Dommel River, enhancing floodwater absorption and creating critical wildlife habitats^32^.

#### Biodiversity conservation

- *Habitat Restoration*: Protect and rehabilitate aquatic and riparian habitats through riverbank reforestation and ecosystem stabilization efforts.
- *Species Recovery Programs*: Conservation actions should focus on the revival of native species, as seen in efforts to restore populations of Gangetic dolphins, otters, and turtles through habitat improvement initiatives in the Ganga River Action Plan^27^.

#### Legal and policy frameworks

- *Strengthening Regulatory Frameworks*: Enhance river management laws and pollution control regulations to ensure effective enforcement.
- *Policy Learning*: Draw lessons from previous programs like the Ganga Action Plan, emphasizing better execution and enforcement, as demonstrated in the Namami Gange Project through continuous monitoring by agencies such as the Central Pollution Control Board (CPCB)^27^.

#### Research and adaptive management

- *Continuous Water Quality Monitoring*: Establish permanent monitoring stations to study long-term impacts of pollution and climate change, drawing inspiration from extensive studies between 2012 and 2022 at key points like Rajghat/Malviya Bridge and Assi Ghat^27^.
- *Smart River Management*: Adopt flexible, data-driven management strategies that incorporate real-time monitoring, artificial intelligence tools, and sensor networks to continuously refine restoration and conservation efforts.

#### Detailed action plan proposed for stretch

Short-term Plans:

- Dredging activities should be undertaken to increase the river’s water-holding capacity, improve flow conditions, and remove obstructions within the river channel. This intervention will enhance flood management and restore more natural river dynamics.
- Public awareness: As the river area, particularly around Manipal Lake, serves as a recreational and picnic spot, public awareness programs should be emphasized through creative approaches: Organizing skit performances to educate visitors and the local community; Designing and installing murals featuring environmental messages; and launching coordinated social media campaigns targeting both residents and tourists to foster a sense of river stewardship.
- A permanent solid waste management system must be established, especially in high-traffic areas like Manipal Lake. Install designated garbage collection points along visitor pathways and picnic spots. Conduct waste characterization studies to understand the types and sources of waste for better management strategies. Immediate measures are needed to prevent the illegal dumping of solid and electronic waste into the river channel through the installation of separate e-waste collection units and public education about proper disposal practices.
- Waste materials should be intercepted and removed by installing proper trapping devices, particularly at locations 06 and 07. These devices will prevent solid waste from entering downstream stretches and help maintain water quality. Existing collapsed structures must be rehabilitated, and any physical obstructions in the flow channel should be promptly addressed to restore natural flow dynamics and prevent localized stagnation.
- To minimize direct disposal of untreated domestic wastewater into the river, decentralized natural treatment systems should be promoted. Encourage the installation of compact filtration units at individual households, utilizing layers of compacted stones and natural filtration media to pre-treat sewage water before discharge.
- Sewage Treatment Plants (STPs) operating within the stretch must undergo a thorough inspection to assess their functionality. Particular attention should be given to facilities contributing to foul odors and elevated pollution levels, and corrective measures must be enforced to ensure compliance with environmental standards.
- Proper embankment protection should be installed along vulnerable river stretches to reduce erosion risks. Native vegetation should be planted along the riverbanks to stabilize soils, enhance biodiversity, and create a natural buffer that filters runoff before it enters the river.

Long-term plans:

- Conduct regular water quality monitoring at intervals of 3–4 months.
- Plant saplings around the lake area and along riverbanks.
- Evict encroached areas along the river to prevent future flooding risks.
- Widen the river channel at location 07 to reduce flow obstruction and flood risks during the monsoon.
- Construct baffle walls at location 08 to promote groundwater recharge near the existing check dam.
- Implement flood management measures at stretch 05, including stormwater pumping units and proper drainage facilities.
- Upgrade and modernize sewage treatment plants (STPs) with updated technologies in consultation with environmental engineering agencies (estimated cost: INR 2–3 crores).
- Install proper waste trapping devices at key locations such as 06 and 07.
- Rehabilitate collapsed structures and remove obstructions in the river channel.
- Promote natural treatment of household sewage using compacted stone filtration units.
- Conduct thorough inspections of all STPs, particularly those contributing to pollution and foul odours.
- Install proper riverbanks and reinforce them with riparian vegetation planting.
- Launch public awareness campaigns using skits, murals, posters, and social media, particularly at picnic and recreational spots like Manipal Lake.
- Establish organized solid waste management systems, including dedicated garbage and e-waste collection points.
- Sequentially record water quality parameters under an assigned monitoring authority.
- Develop walking pathways along both sides of the 14 km river stretch to promote sustainable tourism.
- Construct a coastal reservoir at Malpe to address summer water scarcity for irrigation and drinking water use.

A Cost-Benefit Ratio (CBR) analysis was conducted for each major long-term intervention, specifically: developing STP infrastructure (using nanomembrane technology), periodic water quality monitoring, widening and maintaining river channels, and constructing a coastal reservoir. The approximate costs incurred for each action plan have been compiled. Tables 5, 6, 7 and 8 present the detailed CBR calculations for each intervention. All financial figures are expressed in Indian Rupees (INR). Table 5 outlines the costs associated with the development and modernization of STP infrastructure. Table 6 presents the CBR calculation for implementing real-time water quality monitoring systems. Table 7 details the CBR calculation for widening and maintaining the river channel to improve flow and reduce flood risks. Table 8 provides the CBR calculation for the construction of a coastal reservoir to address water scarcity. Table 3 summarizes and ranks each action plan based on their calculated CBR values to prioritize interventions according to cost-effectiveness.

**Table 5:** Cost-benefit ratio (CBR) calculation for developing and upgrading sewage treatment plant (STP) infrastructure using nanomembrane technology.

Developing STP infrastructure (nanomembrane technology)					
Sl no	Costs incurring	Sum COSTS (INR)	Benefits incurring	Sum benefits (INR)	CBR
1	Capital cost	2,50,00,000	Improved water quality	20,00,000	
2	Operational costs	12,50,000	Energy efficiency	6,50,000	
3	Monitoring and data systems	8,00,000	Reduced pollution	12,00,000	
4	Community engagement and awareness	3,50,000	Compact design	10,00,000	
5			Cost savings	10,00,000	
6			Public health benefits	10,00,000	
	Total	2,74,00,000		68,50,000	0.25

**Table 6:** Cost-benefit ratio (CBR) calculation for implementing periodic and real-time monitoring of water quality parameters along the Indrani river.

Monitoring water quality periodically					
Sl no	Costs incurring	Sum costs (INR)	Benefits incurring	Sum benefits (INR)	CBR
1	Sampling and analysis	1,50,000	Improved water management	12,50,000	
2	Equipment and sensors	4,00,000	Reduced health risks	10,00,000	
3	Labor and Expertise	2,50,000	Ecosystem Preservation	8,50,000	
4	Transportation and Logistics	1,50,000	Policy and Planning	7,00,000	
5	Data management systems	2,50,000	Community engagement	4,00,000	
	Total	12,00,000		42,00,000	3.5

**Table 7:** Cost-benefit ratio (CBR) calculation for widening and maintaining river channels to improve flow capacity and reduce flood risks along the Indrani river.

Widening and maintaining river channels					
Sl no	Costs incurring	Sum costs (INR)	Benefits incurring	Sum benefits (INR)	CBR
1	Widening the river channel	20,00,00,000	Flood prevention	7,50,00,000	
2	Bank stabilization	8,00,00,000	Improved water flow	4,00,00,000	
3	Flood management infrastructure	16,00,00,000	Biodiversity preservation	3,00,00,000	
4	Regular maintenance	1,80,00,000	Tourism boost	2,50,00,000	
5	Nil	Nil	Long-term cost saving	3,00,00,000	
	Total	45,80,00,000		20,00,00,000	0.436681

**Table 8:** Cost-Benefit ratio (CBR) calculation for the creation of a coastal reservoir at Malpe to address seasonal water scarcity for irrigation and drinking water use.

Creating coastal reservoir					
Sl no	Costs incurring	Sum costs (INR)	Benefits incurring	Sum benefits (INR)	CBR
1	Construction costs	6,00,00,00,000	Water security	1,30,00,00,000	
2	Land acquisition	1,30,00,00,000	Flood management	63,00,00,000	
3	Water treatment facilities	70,00,00,000	Economic growth	50,00,00,000	
4	Environmental impact mitigation	63,00,00,000	Environmental Conservation	40,00,00,000	
5	Operational and maintenance costs	15,00,00,000	Improved Public health	25,00,00,000	
6	Nil	Nil	Sustainable urban development	35,00,00,000	
	Total	8,78,00,00,000		3,43,00,00,000	0.390661

Based on the calculations above, periodic monitoring of water quality emerges as the most feasible initial approach, achieving the highest Cost-Benefit Ratio (CBR) value of 3.5. Among the various long-term plans considered, this option also incurs the lowest cumulative cost, making it a financially practical choice. In contrast, the creation of a coastal reservoir, although vital for addressing long-term water scarcity, involves the highest estimated costs due to the significant infrastructure and multiple undertakings required. Establishing priorities based on the CBR analysis is essential for ensuring efficient resource allocation in the restoration efforts for the Indrani River. Water quality monitoring, with a CBR of 3.5, offers substantial benefits relative to its cost, and is thus recommended as the top priority for immediate implementation. Widening and maintaining river channels, although requiring considerable investment, shows a moderate CBR of 0.44 and presents critical benefits such as enhanced flood control and biodiversity conservation. The construction of a coastal reservoir, despite its lower CBR of 0.39, remains a crucial long-term strategy to ensure flood management and water security. Similarly, the development of sewage treatment infrastructure using nanomembrane technology, with a CBR of 0.25, provides specialized advantages, including improved water quality and energy efficiency, but offers less immediate financial return. This analysis recommends initiating the river management strategy with routine water quality monitoring, while simultaneously planning and preparing for the execution of major infrastructure projects to guarantee sustainability and maximize overall environmental and social impact.

#### Floodwater management measures

The Indrani River and its associated network of nallahs (natural drainage channels) play a vital role in regulating the region’s hydrology. These nallahs serve as natural conduits, channeling stormwater runoff from both rural and urban areas into the larger water bodies. However, the drainage systems frequently encounter challenges such as overflow, obstruction, and siltation, which significantly increase the risk of flooding, particularly during the monsoon season.

In Udupi, these issues have become especially severe with the onset of the monsoon season in June, often intensifying in July due to increased rainfall. The resulting problems include the submergence of roads and disruption of transportation networks. To address these challenges, a combination of structural, non-structural, and environmental-based measures has been recommended^33,34^. These approaches can be selectively implemented along different sections of the river depending on the specific site conditions and the severity of the problems encountered.

#### Structural measures

1. Dredging and Excavation for Desilting and Cleaning: As suggested by^34^, the water-carrying capacity of the Indrani River and its tributaries should be maintained by routinely removing accumulated silt and debris through dredging and excavation activities. This practice helps to prevent blockages and ensures uninterrupted water flow.
2. Construction of Embankments and Retaining Walls: Building embankments and retaining walls along the riverbanks and nallahs can effectively prevent the overflow of water into adjacent residential and agricultural areas (Federal Insurance and Administration Risk Management Directorate).
3. Development of Check Dams, Weirs, and Baffle walls: Small check dams, weirs, and baffle walls constructed upstream can regulate the flow of water, reduce flow velocity, and minimize downstream flooding risks. These structures also aid in groundwater recharge.
4. Construction of Retention and Detention Basins: Retention and detention basins should be developed to temporarily store excess rainwater during heavy precipitation events. By releasing water gradually, these basins help to lower peak discharge rates and reduce the risk of sudden flooding^33^.

#### Non-structural measures

1. Zoning of Floodplain areas: Enforce zoning regulations to restrict or control development in high-risk floodplain areas. Proper land-use planning reduces vulnerability to flood hazards and protects natural flood absorption zones.
2. Development of Early Warning Systems: Design and implement early warning systems capable of alerting communities to potential flood events. These systems facilitate timely evacuations, enhance community preparedness, and minimize potential loss of life and property.
3. Community awareness through Social Media Campaigns and Education Programs: Promote public education initiatives, including social media campaigns and awareness programs, to teach the importance of maintaining clear nallahs and adopting flood prevention practices. Engaging the community actively ensures the long-term success of flood management strategies.

#### Environmental and ecosystem-based measures

1. Riparian Buffers: Establish vegetative buffer zones along riverbanks and nallahs to absorb surface runoff, reduce soil erosion, and filter pollutants before they enter the water system. Riparian buffers also provide critical habitat for wildlife and enhance the ecological resilience of the river corridor.
2. Restoration of Wetland: Restore natural wetlands and develop artificial wetlands to act as natural sponges that absorb excess floodwater during heavy rainfall events. Wetlands also improve water quality by trapping sediments, nutrients, and contaminants, contributing to healthier river ecosystems.

Integrated water management plans should be implemented by combining structural and non-structural measures to enhance the effectiveness of flood mitigation strategies. Stormwater management plans and water conservation initiatives must be incorporated to provide efficient, sustainable methods for preserving the ecosystem and mitigating flooding risks in the region. The application of Geographic Information System (GIS) technology is recommended to map flood-prone areas, analyze hydrological data, and support the planning and implementation of effective floodwater management measures.

Based on the assessment of different sections of the river, stretches four and five require immediate attention, as they represent low-lying areas highly vulnerable to flooding due to their topography and hydrological characteristics. Targeted intervention in these stretches is critical to reducing risk. The implementation of integrated management measures in urban areas can significantly reduce stormwater runoff, alleviating pressure on drainage systems. This has important implications for policymakers, legislators, and urban planners, particularly in regions susceptible to flash floods, enabling more resilient and sustainable urban development.

- Structural interventions are required for Stretch Five, including the use of dredgers or excavators for desilting and cleaning operations, along with the construction of embankments and retaining walls. The establishment of retention basins would further assist in recharging groundwater and managing excess stormwater flow in Udupi.
- Geographic Information System (GIS) technology should be employed to map the current land use and land cover (LULC) across the watershed river area. Mapping will help in identifying flood-prone zones and areas contributing significantly to runoff.
- Floodplain mapping should be conducted along stretches Four and Five to delineate areas at higher risk of flooding, thereby aiding in strategic planning for flood mitigation.
- Riverbed clearance is essential in stretches Four and Five to remove accumulated silt and any blockages that hinder the natural flow of water.
- Construction of embankments along the riverbanks in stretches Four and Five is recommended, with embankments raised up to 1 m above the riverbed to accommodate higher water volumes during the monsoon season and reduce flood risks.

As part of the long-term strategy for improving floodwater management in Udupi, the following measures are recommended for implementation.

- Riparian buffer zones should be developed along the riverbanks to prevent soil erosion, stabilize embankments, and enhance the natural resilience of the river system. It is recommended to create two buffering zones to provide additional structural and ecological support.
- Early warning systems should be designed and installed to provide timely alerts for potential flood events, particularly in vulnerable areas such as stretches four and five. These systems will enhance community preparedness and minimize flood-related damages.

**Fig. 9:**
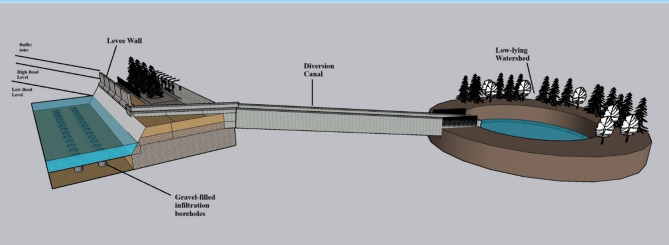
Three-dimensional (3D) model illustrating proposed flood mitigation measures, including levee walls, diversion canals, and gravel-filled infiltration boreholes, developed using SketchUp Pro 2021 software.

There are several key options available for flood mitigation in the low-lying areas of the Indrani River, including the construction of levee walls and diversion canals, combined with gravel-filled infiltration boreholes, as illustrated in Fig. 9.

##### Option 1: levee wall with a combination of gravel-filled infiltration boreholes

In this strategy, a levee wall is constructed along the river in flood-prone short stretches to prevent floodwaters from overflowing into adjacent areas and to guide the natural course of the river. To complement flood control, gravel-filled infiltration boreholes are integrated into the system to promote groundwater recharge during both the summer and monsoon seasons. This dual approach not only mitigates flooding but also addresses seasonal water scarcity issues. The gravel-filled infiltration boreholes are designed with a diameter of 1 foot and are placed at 2-meter intervals along the levee alignment. In the proposed model, low flood levels, high flood levels, and designated buffer zones have been clearly demarcated to assist in effective floodplain management and infrastructure planning.

##### Option 2: diversion canal with gravel-filled infiltration boreholes

In this strategy, a diversion canal is constructed to redirect excess river water into an adjacent low-lying watershed, facilitating temporary storage and groundwater recharge. The canal is equipped with a no-return gate mechanism to control the flow and prevent backflow into the main river channel. As shown in Fig. 9, the diversion canal serves a dual purpose: acting as a rainwater harvesting system and promoting groundwater recharge. Gravel-filled infiltration boreholes, similar to those described in Option 1, are integrated along the canal route to enhance infiltration efficiency. The 3D model presented provides a visual representation of the proposed infrastructure, aiding local authorities in understanding, adapting, and approving the mitigation measures for rapid implementation. The design specifications for the canal, including slope gradients and construction materials, adhere to standard guidelines outlined in the relevant Indian codes of practice. A suitable downstream site must be selected to minimize environmental impacts while maximizing storage and recharge benefits. The introduction of stored floodwaters into low-lying areas is expected to support local ecosystem restoration, enhancing biodiversity by benefiting native flora and fauna. Community engagement is a key component of the approach, drawing inspiration from successful models such as the Namami Gange program, which emphasized public awareness, sustainable practices, and citizen participation. Initiatives like the creation of Ganga Praharis—trained volunteers dedicated to river conservation—serve as effective examples for promoting eco-friendly behavior and preserving riverine ecosystems^27^.

#### Socio-economic factors

Improving the water quality of the Indrani River is vital for ensuring the sustainable availability of water for agriculture, drinking, industrial activities, and ecosystem health. The river plays a central role in the daily lives and economic well-being of city residents by supporting essential water supply functions and maintaining groundwater recharge, which helps reduce the overexploitation of borewells. Ensuring year-round water availability will alleviate many challenges faced by residents, particularly during the summer months when dependence on costly water tankers increases. Despite the financial burden, the quality of tanker-supplied water often remains unreliable. Sustaining and enhancing agricultural productivity, especially among urban and peri-urban farmers, is another significant socio-economic benefit of river restoration. Additionally, frequent flooding and flash floods in downstream areas disrupt daily life during the monsoon season, causing substantial financial losses, health risks, and displacement. These challenges underscore the urgent need for a comprehensive action plan for river management. Furthermore, protecting the river’s biodiversity is crucial to building climate resilience and safeguarding the ecosystem services that benefit present and future generations.

The restoration and sustainable management of the Indrani River directly contribute to achieving key United Nations Sustainable Development Goals (SDGs), particularly SDG 6: Clean Water and Sanitation and SDG 13: Climate Action. By improving water quality, enhancing groundwater recharge, and implementing flood mitigation measures, the project addresses critical targets related to water security, ecosystem protection, and climate resilience. Additionally, maintaining biodiversity and supporting agricultural productivity align with broader objectives under SDG 15: Life on Land, promoting the sustainable use of terrestrial ecosystems for the benefit of current and future generations.

#### Critical reflections on feasibility and risks

Implementing comprehensive action plans for river rejuvenation projects, such as the Indrani River, presents significant challenges. Addressing these challenges is essential for ensuring the long-term sustainability and effectiveness of the interventions.

##### Implementation challenges

One of the main barriers is the lack of institutional capacity at the local level. Municipal bodies and gram panchayats often struggle with manpower and funding issues, making it challenging to maintain infrastructure such as sewage treatment plants (STPs), water traps, and decentralized filtration systems. The adoption of compact natural treatment units at the household level, while promising, may face resistance due to a lack of technical knowledge, concerns about maintenance, and the costs of initial installation. Additionally, the absence of a clearly defined authority to lead the restoration efforts can result in fragmented or duplicated initiatives across different departments.

##### Governance and coordination issues

River management in India generally involves various agencies, including urban local bodies, the Central and State Pollution Control Boards, water resource departments, and district administrations. These agencies have overlapping but poorly defined responsibilities. The absence of an integrated governance mechanism or a central agency with clear authority and accountability often leads to delays in coordination and policy implementation. Additionally, the need for transparent information-sharing and data interoperability between agencies—critical for timely decision-making—remains underdeveloped in most districts.

##### Ecological risks of engineering interventions

Structural measures such as dredging, channel widening, and embankment construction are crucial for restoring hydrological flow and reducing flood risk. However, these measures can also pose ecological risks. Dredging may disrupt aquatic habitats, particularly affecting benthic organisms that are vital for nutrient cycling. Hard engineering solutions, like baffle walls and concrete embankments, can interfere with the river’s natural geomorphology and diminish its connectivity with the floodplain, which impacts fish migration and spawning. Additionally, if the widening of the river channel is not carefully planned, it can alter sediment transport dynamics and lead to increased erosion downstream.

##### Social and economic dimensions

Encroachment eviction, while often necessary, can lead to socio-political resistance, particularly in densely populated or economically disadvantaged areas. To mitigate this, it is essential to implement effective rehabilitation or compensation schemes alongside eviction actions. Additionally, initiatives like rainwater harvesting and solid waste management reforms require changes in community behavior. This transformation can only be achieved through ongoing education and incentive programs. The long-term success of these proposed measures will depend not only on engineering solutions but also on how effectively local communities are involved in monitoring, decision-making, and stewardship.

##### Need for adaptive and participatory approaches

Due to the dynamic nature of river ecosystems and urban growth, static master plans may not be effective. An adaptive management framework is essential, as it includes the flexibility to refine interventions based on continuous monitoring data. The integration of AI-based sensor networks for tracking water quality, as demonstrated in smart city models, can support this objective. Furthermore, participatory governance models that involve local stakeholders, NGOs, and academic experts can create valuable feedback loops to adjust policies and practices accordingly.

## Conclusion

The evaluation of the Indrani River’s water quality, utilizing a combination of quantitative, non-experimental, and exploratory approaches, provides critical insights into the current condition of the river system. This study employed purposive, stratified, and systematic sampling techniques to assess key water quality parameters across 15 strategically selected locations along the river. The findings highlight significant spatial variations in water quality and identify areas requiring immediate and long-term management interventions. Additionally, the study addresses the issue of frequent flooding in the low-lying areas of the Indrani River in Udupi during the monsoon season and proposes appropriate mitigation measures to enhance flood resilience and protect surrounding communities.

According to the study, major sources of pollution along the seven stretches of the Indrani River include solid waste disposal, agricultural runoff, urban runoff—including sewage discharge from residential areas and streets—and inadequacies in waste trapping devices and sewage treatment plants. Pollution issues arising from solid waste disposal, agricultural activities, urban runoff, and direct sewage discharge can be effectively addressed through the short-term action plans outlined in the previous chapter. However, more complex and systemic challenges, such as water scarcity, recurrent flooding, and the need for efficient sewage treatment, require the implementation of long-term strategic interventions to ensure sustainable river management.

Following detailed analysis, results, and spatial assessments, it can be concluded that from Stretch 5 onwards, the Indrani River exhibits higher levels of pollution and is significantly affected by flooding during the monsoon season. Based on in-depth discussions, a combination of short-term and long-term action plans has been recommended to address these challenges. However, effective implementation requires a more detailed feasibility assessment. Local authorities must be engaged and convinced to take the necessary steps toward execution. Additionally, a financial feasibility study is crucial to estimate the overall cost implications of the proposed interventions. Among the recommendations, periodic water quality monitoring stands out as the top priority, with the highest Cost-Benefit Ratio (CBR) of 3.5, offering significant advantages at relatively low expense. Construction of a coastal reservoir (CBR 0.39) and the widening and maintenance of river channels (CBR 0.44) are essential for flood control and water security but are recognized as long-term projects requiring substantial financial investment. The development of sewage treatment infrastructure using nanomembrane technology, with a CBR of 0.25, offers specialized advantages in improving water quality and energy efficiency but yields lower immediate financial returns. A phased approach that begins with the implementation of cost-effective, high-impact strategies such as periodic monitoring, followed by gradual investment in larger infrastructure projects, is therefore recommended to optimize outcomes and ensure the sustainability of river restoration efforts.

As the Indrani River flows over 14.2 km, significant opportunities can be harnessed along its course for sustainable development. Given the recurring issue of water scarcity during the summer months in Udupi, the Indrani River, if properly developed alongside the Swarna River, could provide a more practical and reliable supplementary water source. The potential benefits of rejuvenating the Indrani River are substantial and quantifiable. During field visits, it was observed that sections two and three were reliant on tanker-supplied water due to poor river water quality, which did not meet acceptable standards. Addressing these deficiencies through comprehensive short-term and long-term action plans will not only restore the river’s ecological health but will also significantly benefit the residents of Udupi, particularly during periods of water scarcity. Furthermore, the adoption of artificial recharge methods and flash flood control measures, as proposed, could serve as a model for metropolitan areas facing similar challenges. Implementing such strategies would improve the quality of life, ensure sustainable water management, and enhance the natural flow regime of urban rivers.

## Future scope

For a comprehensive study and analysis of the Indrani River, it is essential to monitor water quality parameters year-round across all seasons. This continuous monitoring would provide a robust framework for understanding the river’s characteristics and support the development of a water quality management plan. It also underscores the need for coordinated efforts among local communities, authorities, and industries to preserve and enhance the river’s health. In addition to standard physicochemical parameters, water samples should be routinely tested for microbial contamination, reflecting growing national and global concerns about microplastic and pathogen pollution. A detailed socio-economic impact analysis of the Indrani River is also critical for addressing the region’s climate resilience, economic sustainability, and environmental conservation goals. This can be achieved through a combination of cost-benefit analysis (CBA), real-time water quality and quantity monitoring, and community-based monitoring initiatives.

At present, there is no available biological data for the Indrani River. Future research should prioritize the collection and analysis of biodiversity indicators, including species richness, habitat quality, and the ecological impacts of pollution on aquatic ecosystems. Furthermore, studies should be expanded to include public health assessments, exploring the relationship between water quality and the prevalence of waterborne illnesses among local populations. While real-time monitoring presents challenges—due to sparse monitoring infrastructure, data gaps, and increasing anthropogenic pressures—addressing these issues is vital. Improved data collection systems, advanced analytical methods, and the establishment of additional, well-distributed ground monitoring stations are necessary to capture critical water quality parameters and support effective river management^35^.

## Data Availability

The datasets generated and/or analyzed during the current study are available from the corresponding author upon reasonable request.
